# GATA Transcription Factor Required for Immunity to Bacterial and Fungal Pathogens

**DOI:** 10.1371/journal.pone.0000077

**Published:** 2006-12-20

**Authors:** Samantha Kerry, Michael TeKippe, Nathan C. Gaddis, Alejandro Aballay

**Affiliations:** Department of Molecular Genetics and Microbiology, Duke University Medical Center Durham, North Carolina, United States of America; Massachusetts General Hospital, United States of America

## Abstract

In the past decade, *Caenorhabditis elegans* has been used to dissect several genetic pathways involved in immunity; however, little is known about transcription factors that regulate the expression of immune effectors. *C. elegans* does not appear to have a functional homolog of the key immune transcription factor NF-κB. Here we show that that the intestinal GATA transcription factor ELT-2 is required for both immunity to *Salmonella enterica* and expression of a C-type lectin gene, *clec-67*, which is expressed in the intestinal cells and is a good marker of *S. enterica* infection. We also found that ELT-2 is required for immunity to *Pseudomonas aeruginosa, Enterococcus faecalis*, and *Cryptococcus neoformans*. Lack of immune inhibition by DAF-2, which negatively regulates the FOXO transcription factor DAF-16, rescues the hypersusceptibility to pathogens phenotype of *elt-2(RNAi)* animals. Our results indicate that ELT-2 is part of a multi-pathogen defense pathway that regulates innate immunity independently of the DAF-2/DAF-16 signaling pathway.

## Introduction

The study of innate immunity received renewed attention when the first parallels between mammalian and *Drosophila melanogaster* immunity were discovered (reviewed in [1]–[3]). Since then, various invertebrate model systems have been used to dissect highly-conserved immune responses without the complications of adaptive immunity. The genetically tractable nematode *Caenorhabditis elegans*, which has been used for decades to study the mechanisms of a number biological processes, has become a well-established invertebrate model for the study of microbial pathogenesis and innate immunity (reviewed in [1], [4]–[7]). *C. elegans* has evolved mechanisms to recognize and respond to potential pathogens using an inducible immune system that contains many highly-conserved effectors, including anti-bacterial proteins, lysozymes, lipases, and C-type lectins [8]. Additionally, conserved signaling pathways have been linked to *C. elegans* immunity, including the CED-3, TGF-β, PMK-1 MAP kinase, and DAF-2 pathways (reviewed in [1], [6], [9]), suggesting that despite the vast evolutionary gulf between nematodes and mammals, some of the underlying mechanisms of immunity may be similar.

Despite the similarities, there are also characteristics of the immune response of *C. elegans* that distinguish it from mammals and other invertebrates. Although Toll receptors, first studied in *Drosophila melanogaster* and subsequently studied in mammals, are highly conserved across species, they do not appear to play a role in *C. elegans* immune responses [10]. Specifically, the single nematode Toll receptor homolog, Tol-1, is expressed in the nematode nervous system and is involved in bacterial avoidance behaviors, rather than in the activation of antimicrobial genes [10], [11]. Consistent with the lack of Toll-like receptor-mediated immunity, there is no evidence of a NF-κB homolog in *C. elegans*. NF-κB-like transcription factors are critical in the regulation of Toll immune responses as they control the expression of key genes involved in innate immunity in many organisms (recently reviewed in [12]). Thus, there is a distinct need to determine exactly which transcription factors are involved in modulating nematode immune responses, some of which may also play important immunity roles in mammals.

Recently, Shapira *et al.* [13] demonstrated that the intestinal transcription factor ELT-2 regulates intestinal immunity to the human pathogen *P. aeruginosa.* Here we show that disruption of ELT-2 causes nematodes to become hypersusceptible to *Salmonella enterica*-mediated killing and that ELT-2 upregulates the expression of *clec-67* which is a marker of *S. enterica* infection in *C. elegans*. Our results also suggest that ELT-2 is part of a multi-pathogen defense pathway that regulates innate immunity not only to *S. enterica* and *Pseudomonas aeruginosa*, but also to the Gram-positive bacterium *Enterococcus faecalis* and to the fungal pathogen *Cryptococcus neoformans*. We also show that ELT-2 functions in *C. elegans* immunity independently of the DAF-2/DAF-16 signaling pathway. These data indicate that ELT-2 is an important regulator of a conserved *C. elegans* immune response to pathogens.

## Results

### elt-2 RNAi animals are hypersusceptible to S. enterica-mediated killing

The digestive tract of *C. elegans* is a primary interface between the immune system and potential bacterial pathogens. This is especially important in the case of bacteria such as the human pathogen *S. enterica* which is capable of establishing a persistent infection in the *C. elegans* intestine [14], [15]. In addition, several known effectors of the *C. elegans* immune system are expressed in the intestinal cells. Because of this link between the intestine and immunity, we sought to identify intestinal transcription factors which might modulate *C. elegans* immune responses.

Over 900 functional transcription factors are predicted to be encoded in the *C. elegans* genome [16]. To identify genes encoding transcription factors involved in the regulation of immune effectors expressed in the *C. elegans* intestinal cells, candidates were selected based on the following criteria: i) genes known to be expressed in the intestine (141 candidates), ii) genes expressed during adulthood (10 of the 141 candidates), and iii) genes which only regulate intestinal functions or for which a function in adulthood has not been reported (7 of the 10 candidates). Of the seven candidates, five were chosen for further analysis based on the availability of RNAi clones [17]. Thus, to address whether the selected candidate genes play a role in *C. elegans* immunity, we used RNAi to inhibit their expression and tested the susceptibility of these gene ablated animals to *S. enterica*. The results shown in Table 1 demonstrate that only RNAi ablation of *elt-2* results in consistent increased susceptibility of *C. elegans* to *S. enterica*.

**Table 1:**
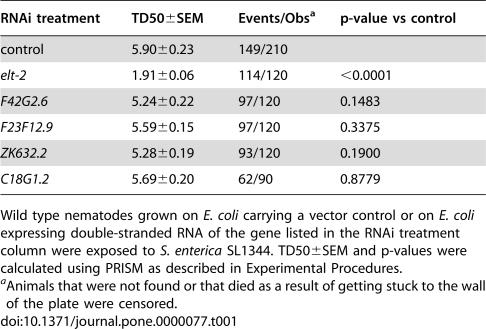
ELT-2 is required for *C. elegans* immunity to *S. enterica*.

RNAi treatment	TD50±SEM	Events/Obs^a^	p-value vs control
control	5.90±0.23	149/210	
*elt-2*	1.91±0.06	114/120	<0.0001
*F42G2.6*	5.24±0.22	97/120	0.1483
*F23F12.9*	5.59±0.15	97/120	0.3375
*ZK632.2*	5.28±0.19	93/120	0.1900
*C18G1.2*	5.69±0.20	62/90	0.8779

Wild type nematodes grown on *E. coli* carrying a vector control or on *E. coli* expressing double-stranded RNA of the gene listed in the RNAi treatment column were exposed to *S. enterica* SL1344. TD50±SEM and p-values were calculated using PRISM as described in Experimental Procedures.

a Animals that were not found or that died as a result of getting stuck to the wall of the plate were censored.

**Figure 1:**
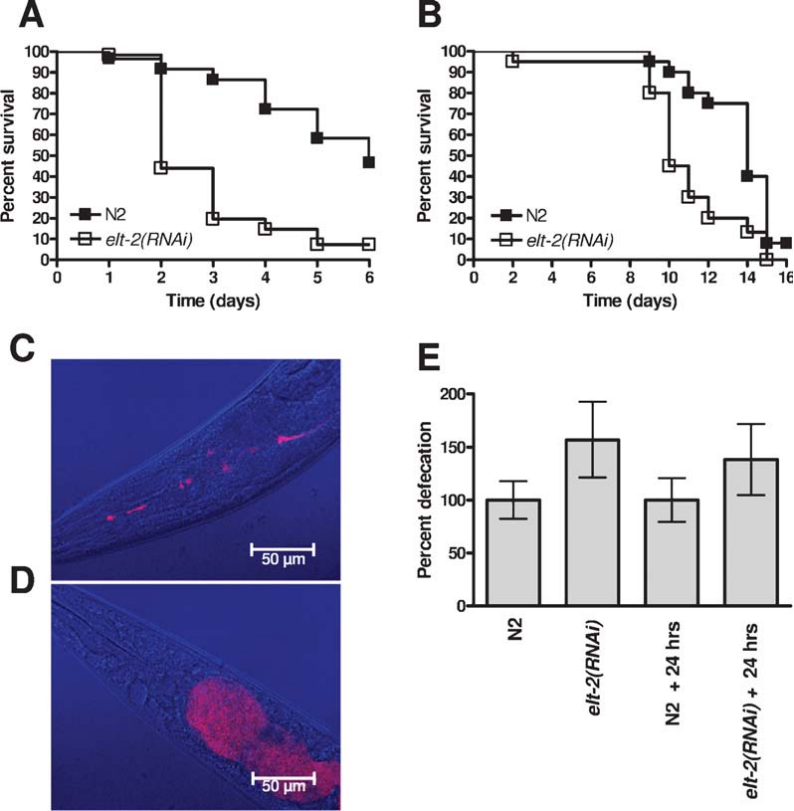
*elt-2*(RNAi) animals are hypersusceptible to *S. enterica-*mediated killing and are colonized by *E. coli*. (**A**) Wild-type nematodes grown on *E. coli* carrying a vector control or on *E. coli* expressing *elt-2* double-stranded RNA were exposed to *S. enterica* SL1344 (P<0.0001). 60 nematodes were used for each condition. Results are representative of at least 3 independent experiments. (**B**) Wild-type nematodes grown on *E. coli* carrying a vector control or on *E. coli* expressing *elt-2* double-stranded RNA were placed on FUdR containing plates with lawns of heat-killed *E. coli* OP50 (P = 0.0011). 20 nematodes were used for each condition. Results are representative of at least 3 independent experiments. (**C**) and (**D**) Wild-type nematodes grown on *E. coli* carrying a vector control (C) or on *E. coli* expressing *elt-2* double-stranded RNA (D) were exposed to *E. coli* expressing DSred for 24 hours, and then visualized using a Leica TCS SL spectral confocal microscope (bar = 50 µm). (**E**) Wild-type nematodes grown on *E. coli* carrying a vector control or on *E. coli* expressing *elt-2* double-stranded RNA were transferred to a clean LB plate either immediately or after 24 hours exposure to *E. coli* OP50 (+24 hrs), where they were allowed to defecate for 2 hours. Plates then were placed at 37°C overnight and colonies counted. The combined data from 10 individual animals are shown, and data were normalized to the median colony count for the vector control at each timepoint. Error bars represent SEM. Results are representative of at least 3 independent experiments.

The increased susceptibility of *elt-2(RNAi)* animals to *S. enterica* (Table 1 and Figure 1A) indicates that ELT-2 may be involved in the regulation of an immune response to this pathogen. However, it is also possible that *elt-2(RNAi)* animals are sickly due to a malfunctioning intestine. ELT-2 is a crucial GATA transcription factor involved in intestinal differentiation [18]–[22] and *elt-2* knockouts exhibit a gut-obstructed (Gob) phenotype that results in arrest at L1 stage larvae and subsequent death, apparently by starvation [18]. Therefore, to study whether *elt-2* ablation by RNAi makes the animals sickly, we first assayed the life span of *elt-2* RNAi nematodes. Figure 1B shows that although *elt-2* RNAi animals exhibit a reduced life span compared to control animals, they survive at least eight days, indicating that starvation is not a cause of death.

To test whether *elt-2(RNAi)* animals are more susceptible to *S. enterica*-mediated killing when taking into account their reduced life span on *E. coli*, the relative mortality of *elt-2(RNAi)* animals feeding on *S. enterica* was used to assess the susceptibility to *S. enterica-*mediated killing of *elt-2* RNAi animals. The relative mortality measure takes into account changes in life span due to a general modification in fitness rather than a specific defect in innate immunity against *S. enterica*. The relative mortality of *elt-2(RNAi)* animals was calculated as described in Materials and methods and determined to be 2.4 (Table 2). The relative mortality obtained is comparable to the relative mortality of animals lacking CED-3-mediated immunity [23] and greater than the relative mortality of animals lacking p38/PMK-1 [24], which is crucial for *C. elegans* immunity [25]–[28], indicating that the shortened life span of the *elt-2(RNAi)* animals infected by *S. enterica* is not simply a consequence of being sickly.

**Table 2:**
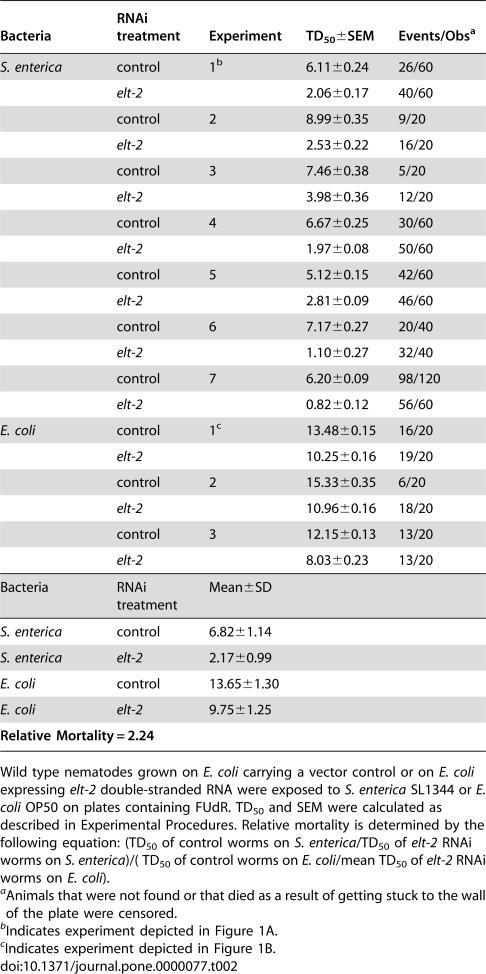
Relative Mortality of *elt-2* RNAi animals on *S. enterica*.

Bacteria	RNAi treatment	Experiment	TD_50_±SEM	Events/Obs^a^
*S. enterica*	control	1^b^	6.11±0.24	26/60
	*elt-2*		2.06±0.17	40/60
	control	2	8.99±0.35	9/20
	*elt-2*		2.53±0.22	16/20
	control	3	7.46±0.38	5/20
	*elt-2*		3.98±0.36	12/20
	control	4	6.67±0.25	30/60
	*elt-2*		1.97±0.08	50/60
	control	5	5.12±0.15	42/60
	*elt-2*		2.81±0.09	46/60
	control	6	7.17±0.27	20/40
	*elt-2*		1.10±0.27	32/40
	control	7	6.20±0.09	98/120
	*elt-2*		0.82±0.12	56/60
*E. coli*	control	1^c^	13.48±0.15	16/20
	*elt-2*		10.25±0.16	19/20
	control	2	15.33±0.35	6/20
	*elt-2*		10.96±0.16	18/20
	control	3	12.15±0.13	13/20
	*elt-2*		8.03±0.23	13/20
Bacteria	RNAi treatment	Mean±SD		
*S. enterica*	control	6.82±1.14		
*S. enterica*	*elt-2*	2.17±0.99		
*E. coli*	control	13.65±1.30		
*E. coli*	*elt-2*	9.75±1.25		
**Relative Mortality = 2.24**				

Wild type nematodes grown on *E. coli* carrying a vector control or on *E. coli* expressing *elt-2* double-stranded RNA were exposed to *S. enterica* SL1344 or *E. coli* OP50 on plates containing FUdR. TD_50_ and SEM were calculated as described in Experimental Procedures. Relative mortality is determined by the following equation: (TD_50_ of control worms on *S. enterica*/TD_50_ of *elt-2* RNAi worms on *S. enterica*)/( TD_50_ of control worms on *E. coli*/mean TD_50_ of *elt-2* RNAi worms on *E. coli*).

a Animals that were not found or that died as a result of getting stuck to the wall of the plate were censored.

b Indicates experiment depicted in Figure 1A.

c Indicates experiment depicted in Figure 1B.

Interestingly, within 24 hours, *elt-2(RNAi)* animals show distended intestines full of *E. coli* that clearly contrast with the non-distended intestinal lumens of control animals (Figures 1C and 1D). This intestinal distension was not seen within 20 minutes of exposure to *E. coli* expressing DSred (data not shown), which indicates that intestinal distension of *elt-2(RNAi)* animals is caused by proliferating *E. coli* rather than by an abnormal intestinal structure. This is not surprising given that proliferating *E. coli* is a cause of death in *C. elegans* [29], that *E. coli* grown on rich media kills *C. elegans* [30], and that immunocompromised animals are killed by *E. coli* [31]. Additionally, after 24 hours of exposure to *E. coli* expressing DSred, *elt-2* RNAi animals exhibited intestinal colonization by the bacteria; DSred fluorescence was present in the *elt-2* RNAi nematode intestine even after three one-hour-transfers onto fresh nonfluorescent *E. coli* plates (data not shown). This indicates that *E. coli* expressing DSred is able to persistently colonize *elt-2* RNAi animals, which is of interest as *E. coli* does not typically colonize the nematode intestine in a persistent fashion [14].

To ensure that the intestinal distension and *E. coli* colonization are not caused by an unseen blockage or an inability of the intestinal muscles to move bacteria through the gut, the defecation of *elt-2(RNAi)* nematodes exposed to *E. coli* was measured. As shown in Figure 1E, defecation of *elt-2(RNAi)* animals is not significantly different from vector control animals. Indeed, while not significantly different, there is a consistent slight increase in the defecation of *elt-2(RNAi)* animals. Taken together, these results indicate that *elt-2(RNAi)* animals do not exhibit the Gob phenotype observed in *elt-2* knockouts, that they do not die by starvation, and that they are immunocompromised animals killed by replicating bacteria capable of colonizing their intestine.

### ELT-2 controls expression of clec-67 which is a marker of C. elegans immune response to S. enterica

To provide further evidence of the role of ELT-2 in innate immunity, we studied its involvement in the expression of the gene *clec-67*, which encodes a C-type lectin. Traditionally, C-type lectins function as soluble mediators that specifically bind carbohydrates, resulting in pathogen clearance by opsonization. A recent report shows that a C-type lectin encoding gene whose product possesses antimicrobial activity is upregulated in Paneth cells by microbial colonization, indicating that lectins are part of a primitive mechanism of innate immunity [32]. In addition, genes encoding C-type lectin domains have been found to be upregulated in *C. elegans* by a human opportunistic pathogen [33], a nematode specific pathogen [34], and a bacterial toxin [28], indicating that they are conserved markers of *C. elegans* immunity.

**Figure 2:**
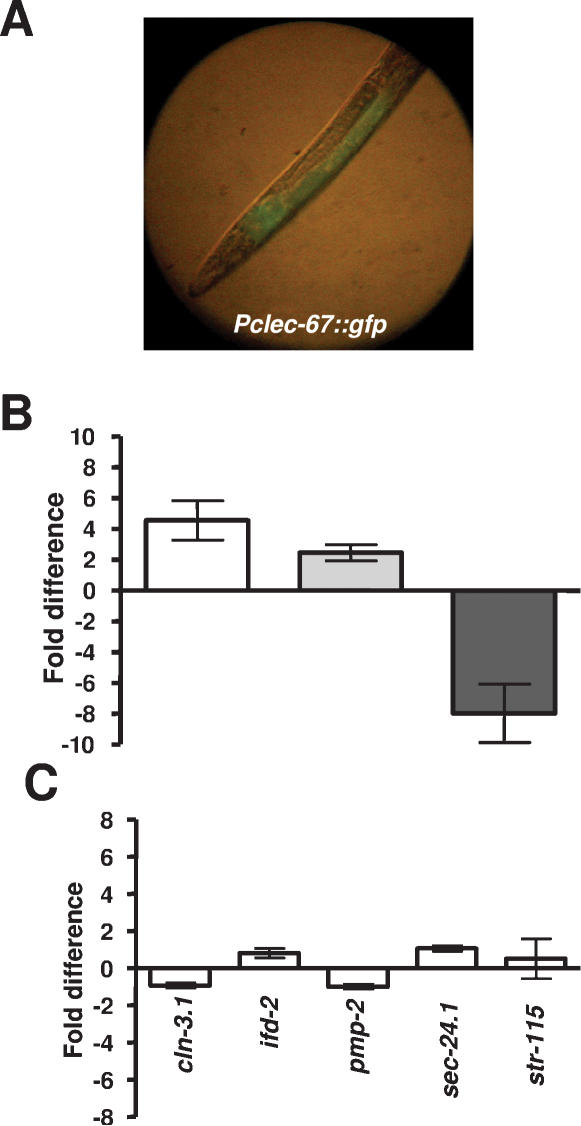
Expression of *clec-67* is controlled by ELT-2. (**A**) *Pclec-67::gfp* nematodes were grown on *E. coli* OP50 and were visualized using a Leica MZ FLIII fluorescence stereomicroscope. (**B**) Microarray (white) and qRT-PCR (light grey) comparing expression of *clec-67* in wild-type nematodes grown on *S. enterica* SL1344 versus wild-type nematodes grown on *E. coli* OP50. qRT-PCR comparing expression of *clec-67* in wild-type nematodes grown on *E. coli* carrying a vector control versus wild-type nematodes grown on *E. coli* expressing *elt-2* double-stranded RNA (dark grey). Results are the average of 3-7 independent experiments. Error bars represent SEM. (**C**) qRT-PCR comparing expression of a variety of intestinal housekeeping genes in wild-type nematodes grown on *E. coli* carrying a vector control versus wild-type nematodes grown on *E. coli* expressing *elt-2* double-stranded RNA. Results are the average of 5 independent experiments. Error bars represent SEM.

We decided to use *clec-67* as a marker of *C. elegans* immunity to *S. enterica* because it is consistently upregulated by this pathogen in several expression profiling experiments performed in our laboratory, because it is expressed in the intestine (Figure 2A), and because its promoter region contains a GATA binding site (A/T GATA A/G). As shown in Figure 2B, the *clec-67* upregulation by *S. enterica* observed in our microarrays was confirmed by qRT-PCR. Figure 2B also shows that when *elt-2* expression is ablated by RNAi, *clec-67* is downregulated compared to vector control, indicating that ELT-2 regulates the expression of this effector of *C. elegans* immunity to *S. enterica*. To ensure that the differences in expression pattern of *clec-67* are not due to intestinal defects in *elt-2(RNAi)* animals, we measured the expression of six intestinal housekeeping genes in *elt-2(RNAi)* and vector control nematodes exposed to *S. enterica* (Figure 2C). The expression of each of these genes is less than two-fold different from vector control, well within the error range of the assay, and much less than the differences seen in expression of *clec-67*. Therefore, the change in the expression pattern of *clec-67* is due to changes in ELT-2 levels, and not to nonspecific changes in intestinal gene expression.

### ELT-2 is required for proper C. elegans immunity to a variety of microbial pathogens

**Figure 3:**
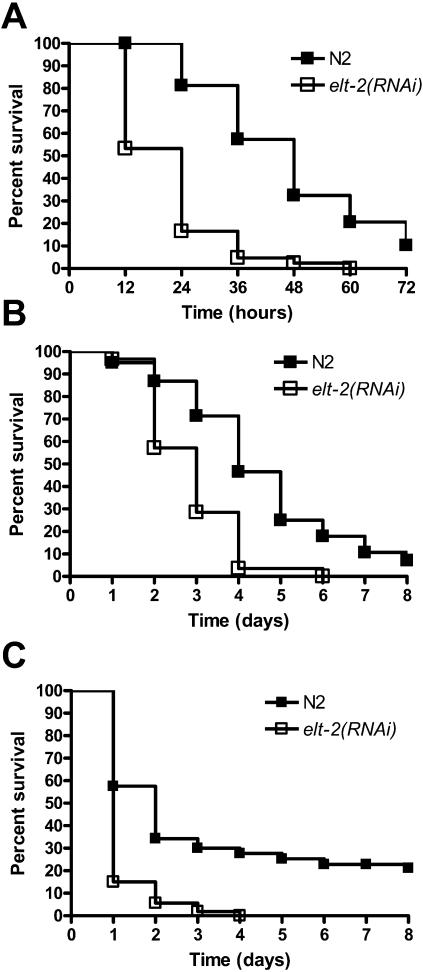
*elt-2(RNAi)* animals are more susceptible than wild-type nematodes to a variety of pathogens. (**A**)Wild-type nematodes grown on *E. coli* carrying a vector control or on *E. coli* expressing *elt-2* double-stranded RNA were exposed to *P. aeruginosa* PA14 (P = 0.0001). (**B**) Wild-type nematodes grown on *E. coli* carrying a vector control or on *E. coli* expressing *elt-2* double-stranded RNA were exposed to *E. faecalis* OG1RF (P<0.0001). (**C**) Wild-type nematodes grown on *E. coli* carrying a vector control or on *E. coli* expressing *elt-2* double-stranded RNA were exposed to *C. neoformans* H99 (P<0.0001). 60–120 nematodes were used for each condition. Results are representative of at least 3 independent experiments.

To determine whether ELT-2 is part of an immune system specific to *S. enterica* or whether it is required for immunity to pathogens in general, *elt-2(RNAi)* animals were infected with the Gram-negative bacterial pathogen *P. aeruginosa* [35], the Gram-positive bacterial pathogen *E. faecalis* [30], and the fungal pathogen *C. neoformans* [36] as described. For all of these pathogens, *elt-2(RNAi)* animals exhibit increased mortality compared to vector control animals (Figure 3). These data indicate that RNAi ablation of *elt-2* increases *C. elegans* susceptibility to different types of bacterial and fungal pathogens.

**Figure 4:**
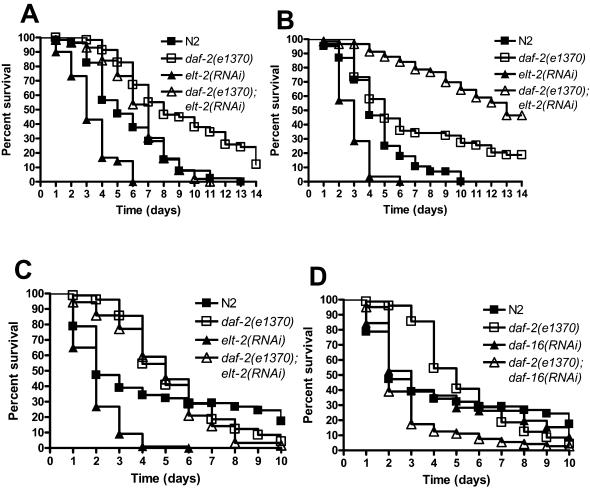
The *daf-2(e1370)* mutation rescues the increased susceptibility to pathogens phenotype of *elt-2(RNAi)* animals. (**A**) Wild-type and *daf2(e1370)* nematodes grown on *E. coli* carrying a vector control or on *E. coli* expressing *elt-2* double-stranded RNA were exposed to *S. enterica* SL1344. Significant differences were found when wild-type was compared to *daf-2(e1370)* (P<0.0001), when *elt-2(RNAi)* was compared to *daf-2(e1370);elt-2(RNAi)* (P<0.0001), and when *daf-2(e1370)* was compared to *daf-2(e1370);elt-2(RNAi)* (P<0.0001). (**B**) Wild-type and *daf2(e1370)* nematodes grown on *E. coli* carrying a vector control or on *E. coli* expressing *elt-2* double-stranded RNA were exposed to *E. faecalis* OG1RF. Significant differences were found when wild-type was compared to *daf-2(e1370)* (P = 0.0056), when *elt-2(RNAi)* was compared to *daf-2(e1370);elt-2(RNAi)* (P<0.0001), and when *daf-2(e1370)* was compared to *daf-2(e1370);elt-2(RNAi)* (P<0.0001). (**C**) Wild-type and *daf2(e1370)* nematodes grown on *E. coli* carrying a vector control or on *E. coli* expressing *elt-2* double-stranded RNA were exposed to *C. neoformans* H99 (P<0.0001). Significant differences were found when wild-type was compared to *daf-2(e1370)* (P = 0.0174) and when *elt-2(RNAi)* was compared to *daf-2(e1370);elt-2(RNAi)* (P<0.0001). No significant difference was found when *daf-2(e1370)* was compared to *daf-2(e1370);elt-2(RNAi)* (P = 0.1666). (**D**) Wild-type and *daf2(e1370)* nematodes grown on *E. coli* carrying a vector control or on *E. coli* expressing *daf-16* double-stranded RNA were exposed to *C. neoformans* H99. A significant difference was found when *daf-2(e1370)* was compared to *daf-2(e1370);daf-16(RNAi)* (P<0.0001), but no significant difference was found when wild-type was compared to *daf-16(RNAi)* (P = 0.9375). 60-240 nematodes were used for each condition. Results are representative of at least 3 independent experiments.

The DAF-2/DAF-16 insulin-like pathway which regulates longevity in *C. elegans* has recently been linked to immunity against bacteria. DAF-16 is a FOXO transcription factor, which regulates a wide variety of genes required for longevity, stress-response, development, and immunity [37], [38], and it is negatively regulated by DAF-2 [39]. Thus, *daf-2* mutants exhibit higher DAF-16 activity and are more resistant to different bacterial pathogens [31], [40], [41]. Because our data indicate that ELT-2 regulates a multi-pathogen immune response, we studied whether the transcription factor is required for the effects of DAF-2 in immunity to pathogens. First, we studied whether *daf-2(e1370)* mutants were more resistant to the fungal pathogen *Cryptococcus neoformans* and then whether RNAi ablation of *elt-2* reduces the resistance phenotype of *daf-2(e1370)* animals. As shown in Figure 4, *daf-2(e1370)* mutants are more resistant not only to *S. enterica* and *E. faecalis*, as previously shown [40], but also to *Cryptococcus neoformans*, indicating that DAF-2 regulates immune response to pathogens in general. Furthermore, this increased resistance to *C. neoformans* is dependent upon DAF-16 function as *daf-2(e1370);daf-16(RNAi)* nematodes have increased mortality when compared to *daf-2(e1370)* mutants (Figure 4D).

To address whether the DAF-2 pathway regulates the immune system of the nematode via ELT-2, RNAi was used to ablate *elt-2* in *daf-2(e1370)* animals which were then exposed to *S. enterica, E. faecalis*, and *C. neoformans*. Visibly, the *daf-2(e1370)*;*elt-2(RNAi)* nematodes appear similar to *elt-2(RNAi)* animals, being thinner, and more transparent than wild-type nematodes or *daf-2(e1370)* mutants. However, the *daf-2(e1370)*; *elt-2(RNAi)* nematodes are significantly more resistant to all pathogens tested than *elt-2* RNAi animals (Figure 4). Interestingly, *daf-2(e1370); elt-2(RNAi)* animals have increased resistance to *E. faecalis* compared to *daf-2(e1370)* animals, indicating that ELT-2 may suppress immune defenses specific to *E. faecalis* that are typically inhibited by DAF-2. Control experiments indicate that *daf-2(e1370);daf-16(RNAi)* animals have increased susceptibility to all pathogens when compared to *daf-2(e1370)* (Figure 4D and data not shown).

## Discussion

Our results suggest that the GATA transcription factor ELT-2 regulates a *C. elegans* innate immunity independently of DAF-2/DAF-16 and that it regulates the expression of *clec-67* which is a marker of *C. elegans* immunity to *S. enterica* infection. Previously, ELT-2 had been linked to intestinal development, but its role in innate immunity was unknown. Since DAF-16 does not appear to be required for immunity to bacterial [40] and fungal pathogens (Figure 4C), the ELT-2 requirement for proper immunity to bacterial and fungal pathogens described here provides one of the first direct evidences indicating that a *C. elegans* transcription factor is required not only for the expression of immunity-related genes but also for survival in the presence of pathogens. Consistent with our results, a paper published while this manuscript was being prepared indicates that ELT-2 is required for immunity to *Pseudomonas aeruginosa* [13].

The most well-known mammalian GATA transcription factor is GATA-3, which regulates the development of CD4+ T cells to a Th2 phenotype [42]. However, since *C. elegans* does not have adaptive immunity, our data indicate that GATA transcription factors are also involved in innate immune responses. This is supported by other invertebrate models, where GATA binding elements have been found in the promoters of genes important in defense responses of *D. melanogaster* [43]–[45], the silkworm *Bombyx mori* [46], and the mosquito *Aedes aegypti* [47]. GATA transcription factors are evolutionarily conserved in a wide variety of eukaryotes and are most often involved in cellular development and differentiation [48]. Human GATAs 4–6, which are the most similar to *C. elegans* ELT-2, are involved in development of gut and cardiac tissue. Additionally, human GATA-4 has been shown to regulate cardiac stress responses [49]–[51], indicating that GATA-4 may have more than a developmental role in mammals.

Since *C. elegans* does not appear to have functional NF-κB-like transcription factors, ELT-2 may be an ancestral transcription factor required for surviving microbial infections in a common ancestor of nematodes and vertebrates. The only other *C. elegans* transcription factor, DAF-16, known to regulate the expression of classical immune effectors, is not required for defense to bacterial pathogens. We show here that like DAF-16, ELT-2 appears to regulate the expression of immune effectors. In addition, it seems to be required for survival in the presence of Gram-negative bacteria, Gram-positive bacteria, and fungal pathogens. Lack of immune inhibition by DAF-2 rescues, fully or partially, the hypersusceptibility to pathogens phenotype of *elt-2(RNAi)* animals suggesting both that *elt-2(RNAi)* animals do not have developmental defects that make them more susceptible to pathogens and that ELT-2 acts upstream or independently of the DAF-2/DAF-16 pathway. The facts that RNAi ablation of ELT-2 results in an increased susceptibility to pathogens and that RNAi ablation of DAF-16 (Figure 4) or mutations in *daf-16* [40] does not affect the susceptibility to pathogens argue in favor of the second possibility. Taken together, our results indicate that DAF-16 and ELT-2 are two key transcription factors that regulate two independent pathways required for *C. elegans* innate immunity to microbial pathogens.

## Materials and Methods

### Microbial and nematode strains

The following strains were used: *Escherichia coli* OP50 [52], *Salmonella enterica* serovar *typhimurium* SL1344 [53], *Enterococcus faecalis* OG1RF [54], *Cryptococcus neoformans* H99 [55], and *Pseudomonas aeruginosa* PA14 [35]. *C. elegans* strains utilized were wild-type N2 and *daf-2(e1370)*. These strains were originally obtained from the Caenorhabditis Genetics Center and maintained in our laboratory.

### Transgenic Animals

To generate the *Pclec-67::gfp* construct, the genomic region 520 to 20 bp upstream of *clec-67* was PCR amplified using the following primers generated by PCR Primer Design for *C. elegans* promoter::gfp fusions (http://elegans.bcgsc.bc.ca/promoter_primers/): TCGTTTCTAATGCTCTCGGAA and TGGGTCCTTTGGCCAATCCCGGGGCGTTTTATGCGGGTTTGTTTA. The *gfp* sequence was amplified from pPD95.79 (Addgene, Cambridge, MA) by PCR with the following primers: CCCGGGATTGGCCAAAGGACCCAAAG and CCGCTTACAGACAAGCTGTGACCG. These PCR products were mixed and an additional PCR with the outside primers was performed to fuse the promoter and *gfp* sequences. The linear PCR product of this reaction was injected at 10 ng/µl into N2 animals to create the transgenic line. The coinjection marker pRF4 was injected at 50 ng/µl. Transgenic animals were anesthetized with 10% sodium azide solution and visualized using a Leica MZ FLIII fluorescence stereomicroscope.

### C. elegans killing assays

*C. elegans* wild-type N2 animals and *daf-2* mutants were maintained as hermaphrodites at 15°C, grown on modified NG agar plates and fed with *E. coli* strain OP50 as described [52]. Cultures were grown in Luria-Bertani (LB) broth and agar plates, except *C. neoformans* H99 and *E. faecalis* OG1RF which were grown in yeast peptone dextrose (YPD) and brain-heart infusion (BHI) medium, respectively. All pathogens were grown at 37°C except *C. neoformans* which was grown at 30°C. Pathogen lawns used for *C. elegans* killing assays were prepared by spreading 10–20 µl of an overnight culture of the bacterial strains on modified NG agar medium (0.35% peptone) in 3.5 cm diameter Petri plates. *C. neoformans* and *E. faecalis* were plated on BHI with 50 µg/ml gentamycin selection. Plates were incubated overnight before seeding them with young adult RNAi animals, created as described above. The killing assays were performed at 25°C and animals were transferred once a day to fresh plates, until no more progeny were evident. Animals were scored at the times indicated and considered dead upon failure to respond to touch.

### C. elegans defecation assays

RNAi animals were transferred individually either directly from RNAi plates (time 0) or after 24 hours of exposure to *E. coli* OP50 on modified NGM plates with 0.35% peptone. Animals were initially individually transferred to clean LB agar plates for 10 minutes to remove excess bacteria, then transferred to a new LB agar plate and allowed to defecate for 2 hours at 25°C. Animals were then removed, and the plates incubated overnight at 37°C. Amount of defecation was determined by counting colonies of *E. coli* on each plate. Data were normalized to the median colony count of vector control for each experiment.

### *C. elegans* aging assays

Modified NGM plates containing 0.35% peptone and 100 µg/ml 5-fluorodeoxyuridine FUdR [56] were plated with *E. coli* OP50 lawns and allowed to incubate at 37°C overnight. Young adult RNAi animals were seeded onto these plates, and scored as indicated for *C. elegans* killing assays. As FUdR inhibits growth of progeny, daily transfer of nematodes was unnecessary.

### RNA interference

We used RNA interference to generate loss-of-function RNAi phenotypes by feeding worms with *E. coli* strain HT115(DE3) expressing double-stranded RNA that is homologous to a target gene [57], [58]. Briefly, *E. coli* harboring the appropriate vectors were grown in LB broth containing ampicillin (100 µg/ml) and tetracycline (10 µg/ml) at 37°C overnight. Bacteria were plated onto NGM plates containing 100 µg/ml ampicillin and 10 mM Isopropyl β-D-thiogalactoside (IPTG) to induce double-stranded RNA expression, and were allowed to grow overnight at 37°C.

Gravid adults were allowed to lay eggs on RNAi-expressing lawns of bacteria for 6-12 hours. The eggs were allowed to develop into young adults on RNAi or vector control plates at 25°C or at 15°C in experiments involving *daf-2(e1370). Unc-22* RNAi was used as a positive control for the creation of loss-of-function phenotypes. Bacterial strains expressing double-stranded RNA to inactivate the *C. elegans* genes have been described [17]. The clone identity was confirmed by sequencing. RNAi-mediated ablation of *elt-2* results in animals that despite being typically smaller and thinner than control counterparts, do survive to adulthood and do not die by starvation (Figure 1B).

### Statistical analyses

Animal survival was plotted as a staircase curve using the PRISM (version 4.00) computer program. Survival curves are considered significantly different than the control when P values are <0.05. Prism uses the product limit or Kaplan-Meier method to calculate survival fractions and the logrank test, which is equivalent to the Mantel-Heanszel test, to compare survival curves. The time for 50% of the nematodes to die (time to death 50, TD_50_) was calculated using Prism software (version 4.01) using a non-linear regression analysis of survival proportions utilizing the equation: Y = Bottom+(Top-Bottom)/(1+10 ^(LogEC^_50_^-X)*Hill Slope)^), where Top is set at 100, Bottom is set at 0, X is the time in days and Y is the percentage of nematodes alive at time X. In this instance, TD_50_ is equivalent to EC_50_. The relative mortality of *elt-2* RNAi animals was calculated using the equation: (TD_50_ of control worms on *S. enterica*/TD_50_ of *elt-2* RNAi worms on *S. enterica*)/(TD_50_ control worms on *E. coli*/TD_50_ of *elt-2* RNAi on *E. coli*).

### Confocal microscopy

Modified NGM plates with 0.35% peptone were plated with overnight cultures of *E. coli* OP50 carrying the pDSred Express plasmid (Invitrogen) and allowed to incubate overnight. Vector control or *elt-2* RNAi young adult nematodes were seeded onto these plates and allowed to feed at 25°C. At the times indicated, the animals were removed from the plates and placed on microscope slides with 2% agarose pads in 10% sodium azide solution. A coverslip was placed on top of the agar pad, and sealed with clear nail polish. Images were captured at the indicated magnifications using a Leica TCS SL confocal microscope and Leica Confocal software (version 2.61 Build 1537) (Leica Microsystems Heidelberg GmbH). Images were resized and adjusted for brightness and contrast in Adobe Photoshop.

### RNA isolation

Gravid adult N2 nematodes were lysed using a solution of sodium hydroxide and bleach, washed, and the eggs synchronized overnight in S basal liquid medium at room temperature. Synchronized L1 animals were seeded onto modified NGM plates with 0.35% peptone containing *S. enterica* or *E. coli* OP50, and incubated at 25°C until the nematodes attained young adult stage (approximately 24–30 hours). The animals were then collected by washing the plates with M9 buffer, and RNA extracted using Trizol reagent. Samples were further purified using a QIAGEN RNeasy kit and residual genomic DNA removed using DNase treatment (DNA-free kit Ambion Inc Austin TX). RNA concentration was determined by spectrophotometer and samples were diluted to 10ng/ul in RNase free water. A minimum of two individual RNA isolations was performed for each experiment.

### Quantitative Real-time PCR (qRT-PCR)

qRT-PCR was conducted using the Applied Biosystems Taqman One-Step Real-time PCR protocol using SYBR Green fluorescence (Applied Biosystems) on an Applied Biosystems 7900HT real-time PCR machine in 96 well plate format. Primers were designed using Aceprimer version 1.2 (http://elegans.bcgsc.bc.ca/aceprimer/aceprimer.shtml). Each independent RNA preparation was measured at least twice by qRT-PCR. Within each experiment, a minimum of duplicate wells was run for every primer set. The data for each gene were normalized to the housekeeping gene, ama-1, the large subunit of *C. elegans* RNA polymerase II. Once normalized, the fold difference between SL1344 and OP50 samples was determined and plotted using PRISM.
